# Synthesis and Biological Evaluation of Fatty Hydrazides of By-products of Oil Processing Industry

**DOI:** 10.4103/0250-474X.54282

**Published:** 2009

**Authors:** S. Toliwal, K. Jadav, K. Patel

**Affiliations:** Department of Industrial Chemistry, Institute of Science and Technology for Advanced Studies and Research (ISTAR), Vallabh Vidyanagar-388 120, India

**Keywords:** Acid oil, oil recovered from spent bleaching earth (ORSBE), oxadiazoles, triazoles, hydrazides, antibacterial, antifungal, streptomycin, immidil

## Abstract

Some new 2-alkyl-5-mercapto-1,3,4-Oxadiazoles and 3-alkyl-5-mercapto-1,2,3-4H triazoles were synthesized from hydrazides of acid oil and oil recovered from spent bleaching earth. These newly synthesized compounds were characterized on the basis of elemental analysis and evaluated for biological properties. Certain derivatives exhibited fairly high antibacterial and antifungal activities when compared with streptomycin and immidil used as standard antibacterial and antifungal agents respectively.

Hydrazides, the acylated derivatives[1] of hydrazine are usually encountered as the simple or monosubsituted (RCONHNH_2_), or as sym-disubsituted (RCONHNHOR) compounds. The latter have been referred to as sec-hydrazides. Besides being useful for a number of biological properties, hydrazides are important starting materials for a wide range of derivatives utilizable in pharmaceutical products and as surfactants. Hydrazides have been known to be associated with antibacterial[2], antifungal[3], anthelmintic[4] and anticonvulsant[5] activities. Various thiosemicarbazide derivatives are reported to possess useful pharmacological properties like, antidepressant[6], antiinflammatory[7] and analgesic[8] activities. In addition to the antibacterial[9] activities exhibited by several triazole derivatives, they are also known for their fungicidal, analgesic and antiinflammatory[10] activities. Oxadiazoles and their derivatives are well known chemotherapeutic agents and their utility has muscle relaxant, and bacteriostatic[11] are well known. Biological assessment of fatty hydrazide and their derivatives has been the focus of earlier investigative studies[12,13].

The present work is carried out with a view to impart value addition targets by-products, acid oil and oil recovered from spent bleaching earth (ORSBE) used for soya bean oil. The fatty hydrazides are further derivatized to obtain new antibacterial and antifungal agents.

The by-products of oil processing industries- acid oil and ORSBE were procured from Ashwin Vanaspti Ltd, Samlaya. Analysis of oils for physicochemical characteristics was tested by standard BIS methods and it gave: sp.gr at 30°, 0.915 and 0.921; acid value, 130.38 and 18.82; iodine value, 115.51 and 123.24; saponification value, 182.75 and 186.01 for acid oil and ORSBE, respectively. The fatty acid composition of oils was determined by gas liquid chromatography (GLC) of methyl esters using capillary column (2 m×0.32 mm) packed with 50% cynopropyl phenyl polysiloxane (BP225) at 220° with nitrogen as carrier gas at flow rate 10 ml/min using FID at an injector temperature of 250° was found to be: palmitic, 12.03 for acid oil and 10.54 for ORSBE; stearic, 10.26 for acid oil and 4.62 for ORSBE; oleic, 36.08 for acid oil and 21.39 for ORSBE; linoleic, 40.01 for acid oil and 52.53 for ORSBE and linolenic, 1.2 for acid oil and 6.09% for ORSBE. All other chemicals used in the study were of laboratory grade and were used without any modification.

Methyl esters of acids from oil were prepared by acid catalyzed esterification using standard method[14]. The esters were purified by distillation under 4-5 mm Hg pressure.

Preparation of fatty hydrazides and their derivatives is schematically represented in Fig 1. For the preparation of fatty acid hydrazides[14] a solution of fatty acid esters (0.1 M) in ethanol (150 ml) was mixed with hydrazine hydrate (95%, 0.2 M) was added. The reaction mixture was refluxed for 3-4 h. It was cooled, and the solid separated was collected, washed and recrystallised from ethanol.

**Fig. 1:**
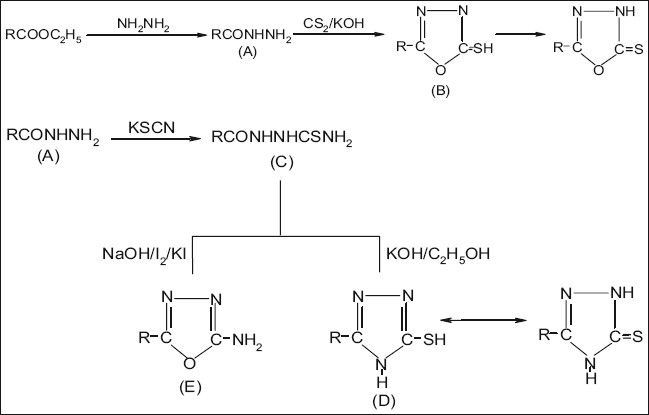
Preparation of fatty hydrazides and their alkyl derivatives. A- Fatty hydrazide, B- 2-alkyl-5-mercapto-1,3,4-oxadiazole, C-thiosemicarbazide, D- 3-alkyl-5-mercapto-1,2,4-4H-triazoles, E-2-alkyl-5-amino-1,3,4-oxadiazole

For the preparation of 2-alkyl-5-mercapto-1,3,4-oxadiazole[15] a solution of a fatty acid hydrazide (0.01 M) in 10 ml of ethanol, a solution of carbon disulfide (2 g) in 3 ml of water and 1 g of potassium hydroxide were refluxed for 7-8 h until the H_2_S is ceased. The contents were cooled and acidified with dilute hydrochloric acid. The separated solid was filtered, collected, washed with water, dried and recrystallised from ethanol.

For the preparation of fatty acid thiosemicarbazide[16] a solution of a fatty acid hydrazide (0.02 M) in methanol (50 ml), potassium thiocynate (0.03 M) and hydrochloric acid 3 ml were mixed added with constant stirring. The mixture was immediately evaporated to dryness on a steam bath and heated for an additional hour with another 50 ml ethanol. The resulting solid was treated with water, little ethanol and recrystallised from ethanol.

For the preparation of 3-alkyl-5-mercapto-1,2,4,-4H-triazoles[17] a solution of thiosemicarbazide (0.01 M) in 15 ml of ethanol, and potassium hydroxide (20 ml, 10%) was refluxed for 7-8 h on a steam bath. It was cooled and acidified with dilute hydrochloric acid to adjust the pH between 5-6. Resulting solid was filtered, dried and recrystallised from ethanol.

For the preparation of 2-alkayl-5-amino-1,3,4-oxadiaazole[14] a solution of thiosemicarbazide (0.01 M) in 15 ml of ethanol was added to a solution of sodium hydroxide (5 ml, 5N) with cooling and stirring. To this clear solution, a solution of I_2_/KI was added till permanent tinge of iodine persisted at room temperature. The mixture was immediately refluxed and more I_2_/KI was added till permanent tinge was obtained. The mixture was then cooled and poured into ice-cold water; the solid that separated was collected by filtration, washed with water and with dilute thiosulfate solution and again with water. The solid was dried and recrystallised from absolute ethanol. The melting point, nitrogen content and percent yield of the compound is given in Table 1.

**TABLE 1:** PHYSICO-CHEMICAL PROPERTIES OF HYDRAZIDES AND THEIR DERIVATIVES

Sample code	Melting point°	Yield %	Nitrogen Content (%)	
			
			Calculated	Found
II_A_	81.0	82.1	9.52	8.68
II_B_	76.8	74.6	11.90	10.34
II_F_	71.8	72.0	8.33	9.24
II_G_	86.9	76.3	12.54	12.55
II_H_	74.5	84.2	13.17	11.78
V_A_	79.3	69.3	9.52	9.89
V_B_	76.1	72.5	11.90	10.86
V_F_	73.2	81.2	8.33	8.94
V_G_	84.7	78.3	12.54	10.58
V_H_	78.1	72.6	13.17	12.18

II_A_- Fatty hydrazide of acid oil, IIB-thiosemicarbazide of acid oil, II_F_-2-alkyl-5-mercapto-1,3,4-oxadiazole of acid oil, II_G_- 3-alkyl-5-mercapto-1,2,4-4H-triazoles of acid oil, II_H_- 2-alkyl-5-amino- 1,3,4-oxadiazole of acid oil, V_A_- fatty hydrazide of ORSBE, V_B_ -thiosemicarbazide of ORSBE, V_F_- 2-alkyl-5-mercapto-1,3,4-oxadiazole of ORSBE, V_G_- 3-alkyl-5-mercapto-1,2,4-4H-triazoles, V_H_- 2-alkyl-5-amino-1,3,4-oxadiazole of ORSBE.

Characterization of synthesized compounds was conformed by IR. Mercaptotriazoles and aminooxadiazoles showed band at 3100 cm^−1^ for NH group. The C=N stretching as observed at 1600 cm^−1^. The mercaptooxadiazoles showed strong band at 1160 cm^−1^ for C=S and absence of band around 2550-2600 cm^−1^. This showed that this compound existed in thione form rather than thiol.

The hydrazides and their derivatives were tested for anti bacterial activity against *Bacillus subtilis* and *Escherichia coli* and antifungal activity against *Aspergillus niger* by agar-agar cup method[18]. Streptomycin and immidil were used as standard antibacterial and antifungal agents respectively.

It can be observed that (Table 1) upon cyclization to thiosemicarbazides, 2-alkyl 5-mercapto 1,3,4 oxadiazoles and 2-alkyl-5-amino-1,3,4-oxadiazoles, the melting points are decreased and are lower than that of the original hydrazide from which they are derived. However, the melting point increase is observed when the fatty hydrazides were cyclised to 3-alkyl-5-mercapto-1,2,4-4H triazoles with latter showing higher melting point than that of the former. This is in agreement with results of the synthesis work of some fatty hydrazide derivatives conducted by Badami *et al.*[19] and by Daulatabad *et al.*[20]. They also reported similar trend of increase in melting point for 3-alkyl 5 mercapto-1,3,4-4H triazoles of different fatty hydrazides and increasing melting points for oxadiazoles of the two fatty hydrazides as compared to respective fatty (oleic and linoleic) hydrazides.

The melting point increase for various hydrazide derivatives was observed in the following order: 3-alkyl-5-mercapto-1,2,4-(4H)-triazoles>2-alkyl-5-amino-1,3,4-oxadiazoles>2-alkyl-5-mercapto-1,3,4-oxadiazoles. This result is also in agreement with the results obtained by Badami *et al.*[19] and Daulatabad *et al.*[20] for corresponding fatty derivatives.

The results of antibacterial activities of hydrazides and their derivatives (Table 2) highlights following points: When tested for antibacterial activity against *Bacillus subtilis* and *Escherichia colid* acid oil-based derivatives namely thiosemicarbazide, 2-alkyl-5-mercapto-1,3,4-oxadiazole, 3-alkyl-5-mercapto-1,2,4-4H-triazoles, 2-alkyl-5-amino-1,3,4-oxadiazole, exhibited mild (0-25%) activities. ORSBE-based derivatives namely thiosemicarbazide, 2-alkyl-5-mercapto-1,3,4-oxadiazole exhibited fair (25-50%), while hydrazides, 2-alkyl-5-amino-1,3,4-oxadiazole of ORSBE a showed poor and 3-alkyl-5-mercapto-1,2,4-4H-triazoles demonstrated mild (0-25%) acvtivities against *Bacillus subtilis* relative to streptomycin used as standard. ORSBE based derivatives namely hydrazide, 2-alkyl-5-mercapto-1,3,4–oxadiazole exhibited mild (0-25%), while thiosemicarbazide, 3-alkyl-5-mercapto-1,2,4-4H-triazoles, 2-alkyl-5-amino-1,3,4-oxadiazole demonstrated poor bacterial growth retardation against *Escherichia coli* relative to streptomycin used as standard.

**TABLE 2:** ANTIBACTERIAL ACTIVITIES OF ACID OIL AND ORSBE HYDRAZIDES AND THEIR DERIVATIVES

	Bacillus subtilis				*Escherichia coli*			
		
Sample code	Zone standard 200 μg (mm)	Zone sample 200 μg (mm)	Control	growth %	Zone standard 200 μg (mm)	Zone sample 200 μg (mm)	Control	growth %
II_A_	---	−Ve	---	---	23	−Ve	---	---
II_B_	22	4.0	---	+	23	1.2	---	+
II_F_	23	4.4	---	+	22	1.2	17	+
II_G_	22	3.2	---	+	22	0.8	17	+
II_H_	24	2.4	---	+	26	1.2	16	+
V_A_	−Ve	−Ve	---	---	24	0.8	16	+
V_B_	20	7.2	---	++	23	−Ve	---	---
V_F_	23	6.4	---	++	26	0.4	18	+
V_G_	21	4.8	---	+	21	−Ve	16	---
V_H_	−Ve	−Ve	---	---	20	−Ve	17	---

II_A_- Fatty hydrazide of acid oil, II_B_- thiosemicarbazide of acid oil, II_F_ - 2-alkyl-5-mercapto-1,3,4-oxadiazole of acid oil, II_G_- 3-alkyl-5-mercapto-1,2,4-4H triazoles of acid oil, II_H_- 2-alkyl-5-amino-1,3,4-oxadiazole of acid oil, V_A_- fatty hydrazide of ORSBE, V_B_- thiosemicarbazide of ORSBE, V_F_ -2-alkyl-5-mercapto-1,3,4-oxadiazole of ORSBE, V_G_- 3-alkyl-5-mercapto-1,2,4-4H-triazoles, V_H_- 2-alkyl-5-amino-1,3,4-oxadiazole of ORSBE. + = 0-25%, ++ = 25-50%, +++ = 50-75%, ++++ = 75-100%, +++++ = More than 100%.

The results of antifungal activities of acid oil and ORSBE hydrazides and their derivatives (Table 3) highlight following points: when tested for antifungal activity against *Aspergillus niger* acid oil based 2-alkyl-5-amino-1,3,4-oxadiazole, ORSBE-based hydrazide and 2-alkyl-5-amino- 1,3,4-oxadiazole showed mild (0-25%) antifungal activity, while 3-alkyl-5-mercapto-1,2,4-4H triazoles of acid oil, 2-alkyl-5-mercapto-1,3,4-oxadiazole of ORSBE exhibited good (50-75%) fungal growth retardation. The hydrazide, thiosemicarbazide and 2-alkyl-5-mercapto-1,3,4-oxadiazole of acid oil, thiosemicarbazide and 3-alkyl-5-mercapto-1,2,4-4H triazoles of ORSBE, showed poor antifungal growth against *Aspergillus niger* relative to immidil used as a standard.

**TABLE 3:** ANTIFUNGAL ACTIVITIES OF ACID OIL AND ORSBE HYDRAZIDES AND THEIR DERIVATIVES

Sample code	Zone standard 100ppm (mm)	Zone sample 100ppm (mm)	Control	growth %
II_A_	---	---	---	---
II_B_	---	−Ve	---	---
II_F_	---	−Ve	---	---
II_G_	13	7.0	---	+++
II_H_	13	4.0	---	+
V_A_	13	2.0	---	+
V_B_	---	−Ve	---	---
V_F_	13	8.0	---	+++
V_G_	---	−Ve	---	---
V_H_	13	2.0	---	+

II_A_- Fatty hydrazide of acid oil, II_B_- thiosemicarbazide of acid oil, II_F_ - 2-alkyl-5-mercapto-1,3,4-oxadiazole of acid oil, II_G_- 3-alkyl-5-mercapto-1,2,4-4H triazoles of acid oil, II_H_- 2-alkyl-5-amino-1,3,4-oxadiazole of acid oil, V_A_- fatty hydrazide of ORSBE, V_B_- thiosemicarbazide of ORSBE, V_F_ -2-alkyl-5-mercapto-1,3,4-oxadiazole of ORSBE, V_G_- 3-alkyl-5-mercapto-1,2,4-4H-triazoles, V_H_-2-alkyl-5-amino-1,3,4-oxadiazole of ORSBE. + = 0-25%, ++ = 25-50%, +++ = 50-75%, ++++ = 75-100%, +++++ = More than 100%.

It can be concluded that thiosemicarbazide and 2-alkyl-5-mercapto-1,3,4-oxadiazoles of ORSBE can be used as antibacterial agent against *Bacillus subtilis* due to their fair antibacterial activities (25-50%) whereas 3-alkyl-5-mercapto-1,2,4-4H-triazoles of acid oil, and 2-alkyl-5 mercapto-1,3,4-oxadiazole of ORSBE exhibited good (50-75%) fungal growth retardation against *Aspergillus niger* due to their fairly high antifungal activities.
